# Coexistence of Essential Thrombocythemia and Waldenström Macroglobulinemia: A Case Report

**DOI:** 10.1155/crh/3390770

**Published:** 2025-08-28

**Authors:** Meghan Wallace, Bruce Mathey, Cecilia C. S. Yeung, Jacob S. Appelbaum, Mark Wallace

**Affiliations:** ^1^Department of Family Practice, University of Colorado, Denver, Colorado, USA; ^2^Department of Hematology-Oncology, Skagit Regional Health, Mount Vernon, Washington, USA; ^3^Department of Pathology, Fred Hutchinson Cancer Center, Seattle, Washington, USA; ^4^Department of Hematology-Oncology, Fred Hutchinson Cancer Center, Seattle, Washington, USA; ^5^Department of Internal Medicine, Skagit Regional Health, Mount Vernon, Washington, USA

## Abstract

Waldenström macroglobulinemia (WM) and essential thrombocythemia (ET) are distinct hematologic malignancies that have only been reported to co-occur in one previous patient. We present a 64-year-old man with a significant family history for WM who was found to have both ET and WM. He had symptomatic ET, diagnosed by elevated platelets and a positive JAK2 V617F mutation, and asymptomatic WM was found on serum electrophoresis done for screening due to family history. Genomic evaluation of the myeloid and lymphoid cells suggested independent neoplastic transformation. This is the second reported case of a patient with both WM and ET. There was no evidence for a shared mechanism in these dual malignancies.

## 1. Background

Essential thrombocythemia (ET) is a BCR-ABL1-negative myeloproliferative neoplasm (MPN). Polycythemia vera and primary myelofibrosis are other BCR-ABL1-negative MPNs commonly considered in the differential diagnosis of ET. These neoplasms typically exhibit terminal myeloid cell expansion in the blood [1]. ET is rare, with an incidence of 0.2–2.5 new cases per 100,000 population per year. Acquired somatic mutations causing JAK-STAT pathway upregulation are associated with ET, specifically JAK2, CALR, or MPL mutations. Though typically sporadic, familial cases have been documented [2].

Waldenström macroglobulinemia (WM) is a lymphoblastic lymphoma characterized by a clonal proliferation of B cells with plasmacytic differentiation, which leads to the production of IgM monoclonal protein. It is also rare, with an incidence of about 3 per 1,000,000 per year [3]. WM is also typically sporadic, but up to 20% of cases are familial, and diagnosis has been found to be 15.8-fold higher among first-degree relatives as compared to the general population [4].

There have been a few reported cases of ET or other MPNs co-occurring with plasma cell neoplasms, such as monoclonal gammopathy of undetermined significance (MGUS) of non-IgM type or multiple myeloma (MM), but only one case of ET with concomitant WM [5]. We report the 2^nd^ known case of ET and WM.

## 2. Case Presentation

A 64-year-old Caucasian male was evaluated for an 8-month history of headaches, noncardiac chest pain, and fatigue. He reported no associated fevers, chills, or weight loss. His past medical history was positive for paroxysmal atrial fibrillation (PAF) and autoimmune thyroiditis. He had no history of arterial or venous thromboses or clinically significant bleeding. His family history was positive for WM in his father, paternal uncle, and one paternal cousin, as well as MM in another paternal uncle and a paternal cousin. Two additional paternal cousins are being followed for monoclonal gammopathies. A brother has ET. The patient had never smoked and drank alcohol occasionally. He had extensive exposure to herbicides and pesticides as a young man.

The physical exam was unremarkable. Vital signs were normal. The patient was a nontoxic appearing gentleman in no distress. He had no scleral icterus, splenomegaly, hepatomegaly, rash, or palpable lymphadenopathy. An abdominal ultrasound was unremarkable.

A complete blood count (CBC) initially disclosed an elevated platelet count of 743 × 10^9^/L. A CBC two weeks later revealed a platelet count of 634 × 10^9^/L, hemoglobin of 13.2 g/dL, and WBC of 9.3 × 10^9^/L. Due to the elevated platelet count, a JAK2 V617F mutation test was performed and found to be positive, providing supportive evidence of a diagnosis of ET. Serum protein electrophoresis was checked because of the family history of WM, revealing an M spike of 0.5 g/dL, an IgM of 537 mg/dL, IgG of 945 mg/dL, beta globulin of 0.9 g/dL, and albumin of 3.8 g/dL. Six months later, his free kappa light chains were 24.4 mg/L, free lambda light chains were 12.3 mg/L, and kappa/lambda ratio was 1.98; this was the first measurement of free light chains. Peripheral blood smear at diagnosis revealed mild thrombocytosis.

**Figure 1:**
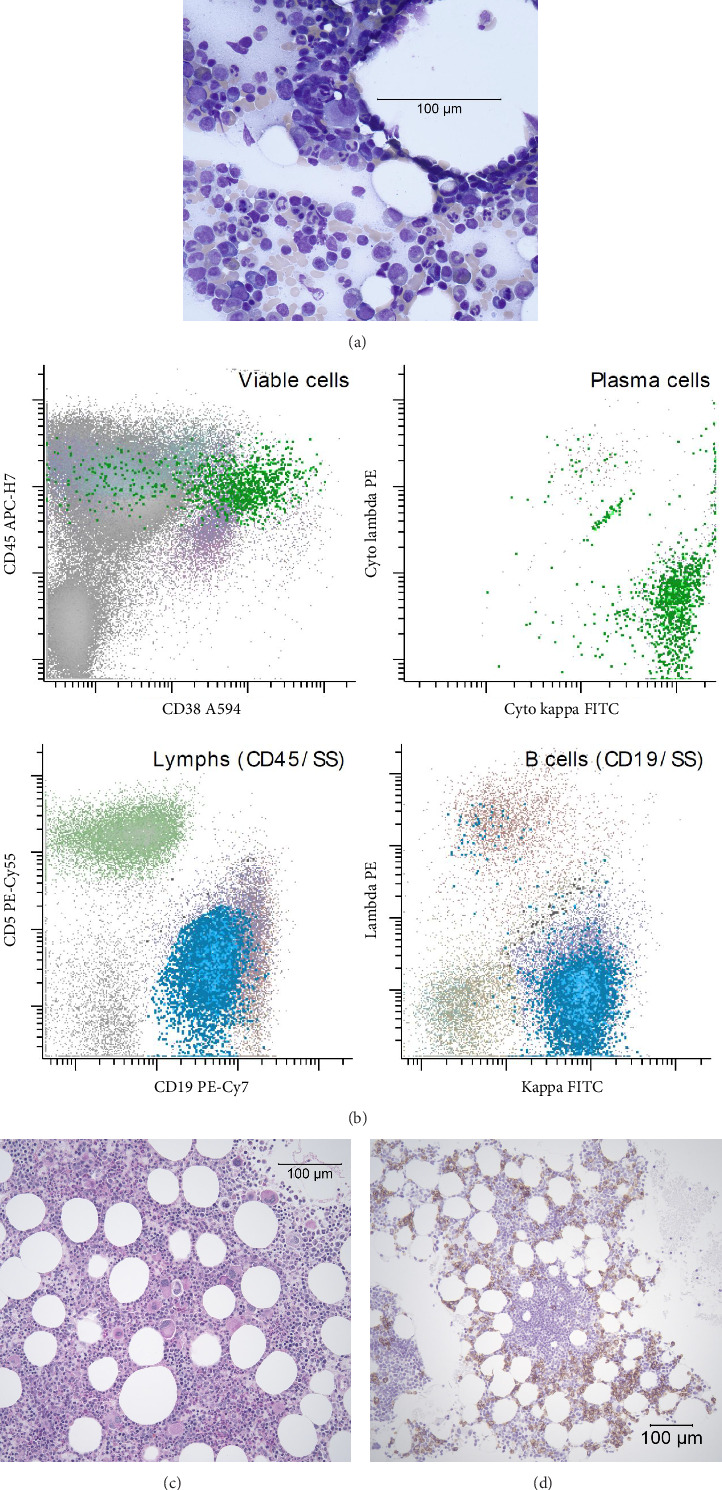
Images from the third and most recent marrow study from our patient. (a) A Wright–Giemsa–stained marrow aspirate featuring a small hyperlobulated micromegakaryocyte with scattered plasma cells (image taken at 40 ×). (b) Four select flow cytometry panels that demonstrate kappa monoclonal B cells and plasma cell populations. (c) A PAS stain particle preparation with a marrow particle featuring increased small hypolobated megakaryocytes which are clustering (image taken at 20 ×). (d) Immunohistochemistry staining for CD138 highlighting a lymphoid aggregate in the center comprised of CD138 negative small lymphocytes being surrounded by CD138 positive plasma cells; note the percentage of plasma cells in this field is increased although the overall plasma cell percentage was ∼10%–20% (image taken at 10 ×).

In total, the patient had three marrow biopsies. The original diagnostic marrow performed at initial diagnosis showed predominant features of a MPN with confirmation of a JAK 2 mutation. A small IgM kappa M spike was detected by serum protein electrophoresis with concurrent small kappa restricted plasma cell population of less than 5% by flow cytometry of bone marrow. No significant reticulin staining was noted. A subsequent marrow study conducted 11 months later demonstrated a hypospicular aspirate and an inadequate core biopsy. A third marrow aspirate and biopsy conducted 11 months after the second showed mildly hypercellular marrow (50% cellularity) with increased atypical megakaryocytes which were clustering, including many small hypolobated forms, micromegakaryocytes, and some larger forms. There were mildly increased plasma cells (see Figures 1(a) and 1(c)). There were lymphoid aggregates comprised primarily of small mature appearing lymphocytes in a nonparatrabecular location with scattered increased plasma cells. Immunohistochemistry for CD34 showed < 5% blasts and CD138 confirmed increased plasma cells ∼10%–20% (see Figure 1(d)). Flow cytometry revealed abnormal, kappa-restricted B cells and plasma cells (see Figure 1(b)). Molecular testing detected a MYD88 L265P mutation. B-cell depleted, and plasma cell depleted DNA was obtained and subjected to sequencing for MYD88, which showed no MYD88 but did show a JAK2 V617F mutation at a VAF of 5%. Marrow aspirate using unsorted cells identified both MYD88 L265P and JAK2 mutations; ATM was negative. The final pathology diagnosis was consistent with a JAK2 positive chronic MPN and a B cell lymphoma with plasmacytic differentiation, consistent with WM. Given the absence of splenomegaly or reticulin staining, the hematopathologist and oncologist concluded that the diagnostic criteria for both ET and WM had been satisfied.

The patient was started on hydroxyurea at doses ranging from 500 to 1000 mg per day and continued taking 325 mg of aspirin daily, which he had been prescribed for PAF. On this treatment, his headaches and atypical chest pain resolved, and his fatigue improved. Platelet counts steadily decreased. After 3 months of treatment, his platelet count was 370 × 10^9^/L, near the goal of 350 × 10^9^/L, and remained stable with occasional hydroxyurea dose adjustments over the following 60 months. The patient's WM continues to be asymptomatic (smoldering WM) though his hemoglobin has slowly declined from 14.5 to 11.7 g/dL, his hematocrit has dropped from 42% to 35%, and his IgM has steadily increased from 500 to 2780 mg/dL. Kappa light chains have risen from 24.4 to 42.5 mg/dL while lambda chains have been stable at 12 mg/dL, resulting in the kappa/lambda ratio increasing from 1.98 to 3.83.

## 3. Discussion and Conclusions

The only other reported patient with both ET and WM is a 55-year-old Chinese male who was found to have an asymptomatic thrombocytosis. That patient also had no lymphadenopathy, hepatomegaly, or splenomegaly. He had no significant medical history, aside from pleural tuberculosis treated 2 years prior, and family history was not mentioned. His serum immunoglobulin analysis revealed an elevated IgM of 2150 mg/dL, kappa light chain of 26.8 g/L, and lambda light chain of 5.03 g/L. His IgM was confirmed to be monoclonal on immunofixation. Bone marrow biopsy showed mild hypercellularity (55%) and excess lymphocytes and large, mature megakaryocytes with hyperlobated nuclei. Bone marrow aspirate showed excess platelets and lymphoplasmacytoid lymphocytes. Flow cytometry confirmed monoclonal B lymphocytes, positive for CD19, CD20, and cell membrane kappa light chain. He had JAK2 V617F, MYD88 L265P, and ATM F1036L genetic mutations found on a 126-mutation panel. He was started on pegylated interferon and 100 mg aspirin daily. His platelet count was 324 × 109/L and IgM level was 1180 mg/dL after 2 months on this therapy [5]. Overall, his case is remarkably similar to our patient's. The apparent response of both his malignancies to interferon therapy is intriguing. Interferon is another agent of value in the treatment of ET and has been shown to have some activity in WM [6].

Our patient has instead been treated with aspirin and hydroxyurea for ET, which has a favorable side effect profile as compared to interferon [7]. His WM is best described as smoldering WM, similar to the more common IgM MGUS in that there is a monoclonal IgM spike but no significant anemia or symptoms of tumor mass/infiltration. The key difference is that smoldering WM has ≥ 10% bone marrow infiltration and a propensity to progress to full blown WM [8]. Smoldering WM is usually observed and not treated to avoid complications of treatment, such as prolonged immunosuppression as with rituximab or development of more aggressive leukemias or lymphomas with alkylating agents or nucleoside analogs [9].

Synchronous dual hematologic malignancies (SDHMs) are unexpectedly found in 1.5% of new patients evaluated for a primary hematologic malignancy [10]. Given the rarity of these SDHMs, there is little data on their optimal management. While there have been only 2 cases of ET with coexistent WM, there have been several reports of MPNs occurring with MM, MGUS of non-IgM type, or IgM MGUS. Typically, when an MPN and smoldering MM or MGUS are both identified, therapy targets the MPN, and the asymptomatic process is observed. If a patient develops overt MM, therapy is swapped, and the MM is treated while the MPN is monitored. In a study of 15 SDHM patients over a 5-year period, 2 patients developed lymphoid leukemia, 1 developed lymphoma, and 1 developed acute myeloid leukemia (AML), raising the concern that patients with coexisting myeloid and lymphoid-derived neoplasms are prone to leukemic or lymphomatous transformation [11].

Given our patient's documented multigenerational family history of WM and MM, a common exposure or genetic predisposition is possible. Some, but not all studies have found an association between pesticide exposure and MPNs, but we did not identify consistent common exposures within the family cohort; three of the six family members with plasma cell malignancies had heavy farm chemical exposures but the other 3 had no such pesticide history [12, 13]. However, it is well known that there is a high degree of clustering for B-cell disorders among first-degree relatives of patients with WM [14].

Both WM and ET are rare diseases. Their co-occurrence in a single patient raises the possibility of a shared mechanism in the process of neoplastic transformation. Genetic mutations that predispose for myeloproliferative diseases mirror mutations seen in sporadic MPNs including JAK2, MPL, or CALR, as well as genes associated with hereditary erythrocytosis and thrombocytosis (EPOR, VHL, HIF1a, and HIF2a) and rare variants in TERT, GRb, ATM, CCDC26, and the JAK2 46/1 haplotype [15]. The L265P mutation found in this patient has been associated with WM, as well as other lymphoid malignancies [16, 17]. The previously reported case of concurrent WM and ET also expressed JAK2 V617F and MYD88 L265P mutations, but separate genomic evaluation of myeloid and lymphoid cells was not conducted as was done in this case. The distinct mutation profile of the two cell lineages in this case (i.e., MYD88 L265P mutated lymphoid cells and JAK2 V617F mutated myeloid cells) suggests independent neoplastic transformation. In a small series of cases, no instance of germline hotspot mutations in MYD88 was identified, suggesting that the MYD88 mutation arises in a B-cell restricted lineage [18]. Therefore, we conclude that while heritable risk factors contributed to the development of WM, it was most likely separate mutational events that led to the development of ET and WM in this patient.

Further investigation is warranted to better understand possible shared mechanisms or genetics of these two diseases.

## Data Availability

The authors declare that all data regarding this case are available within the article.

## Nomenclature

AML: Acute myeloid leukemia
CBC: Complete blood count
DLBCL: Diffuse large B-cell lymphoma
ET: Essential thrombocythemia
IgM: Immunoglobulin M
MGUS: Monoclonal gammopathy of undetermined significance
MM: Multiple myeloma
MPN: Myeloproliferative neoplasm
PAF: Paroxysmal atrial fibrillation
SDHMs: Synchronous dual hematologic malignancies
WM: Waldenström macroglobulinemia

## References

[1] Dickstein J. I., Vardiman J. W. (1995). Hematopathologic Findings in the Myeloproliferative Disorders. *Seminars in Oncology*.

[2] Accurso V., Santoro M., Mancuso S. (2020). The Essential Thrombocythemia in 2020: What We Know and Where We Still Have to Dig Deep. *Clinical Medicine Insights: Blood Disorders*.

[3] Groves F. D., Travis L. B., Devesa S. S., Ries L. A., Fraumeni J. F. (1998). Waldenström’s Macroglobulinemia: Incidence Patterns in the United States, 1988–1994. *Cancer*.

[4] Royer R. H., Koshiol J., Giambarresi T. R., Vasquez L. G., Pfeiffer R. M., McMaster M. L. (2010). Differential Characteristics of Waldenström Macroglobulinemia According to Patterns of Familial Aggregation. *Blood*.

[5] Lu N., Neoh C. L., Ruan Z. (2020). Essential Thrombocythaemia with Concomitant Waldenström Macroglobulinaemia: Case Report and Literature Review. *OncoTargets and Therapy*.

[6] Legouffe E., Rossi J. F., Laporte J. P. (1995). Najman A Treatment of Waldenstrom’s Macroglobulinemia With Very Low Doses of Alpha Interferon. *The Leukemia & Lymphoma Society*.

[7] Quesada J. R., Talpaz M., Rios A., Kurzrock R., Gutterman J. U. (1986). Clinical Toxicity of Interferons in Cancer Patients: A Review. *Journal of Clinical Oncology*.

[8] Gertz M. A., Fonseca R., Rajkumar S. V. (2000). Waldenström’s Macroglobulinemia. *The Oncologist*.

[9] Leleu X., Soumerai J., Roccaro A. (2009). Increased Incidence of Transformation and Myelodysplasia/Acute Leukemia in Patients With Waldenström Macroglobulinemia Treated With Nucleoside Analogs. *Journal of Clinical Oncology*.

[10] Kotchetkov R., Ellison E., McLean J., Pressnail B., Nay D. (2018). Synchronous Dual Hematological Malignancies: New or Underreported Entity?. *Hematology*.

[11] Malhotra J., Kremyanskaya M., Schorr E., Hoffman R., Mascarenhas J. (2014). Coexistence of Myeloproliferative Neoplasm and Plasma-Cell Dyscrasia. *Clinical Lymphoma, Myeloma and Leukemia*.

[12] Duncombe A. S., Anderson L. A., James G. (2020). Modifiable Lifestyle and Medical Risk Factors Associated With Myeloproliferative Neoplasms. *Hema Sphere*.

[13] Kokouva M., Bitsolas N., Hadjigeorgiou G. M., Rachiotis G., Papadoulis N., Hadjichristodoulou C. (2011). Pesticide Exposure and Lymphohaematopoietic Cancers: A Case-Control Study in an Agricultural Region (Larissa, Thessaly, Greece). *BMC Public Health*.

[14] Treon S. P., Hunter Z. R., Aggarwal A. (2006). Characterization of Familial Waldenström’s Macroglobulinemia. *Annals of Oncology*.

[15] Bellanné-Chantelot C., Rabadan Moraes G., Schmaltz-Panneau B., Marty C., Vainchenker W., Plo I. (2020). Germline Genetic Factors in the Pathogenesis of Myeloproliferative Neoplasms. *Blood Reviews*.

[16] Treon S. P., Xu L., Yang G. (2012). MYD88 L265P Somatic Mutation in Waldenström’s Macroglobulinemia. *New England Journal of Medicine*.

[17] Jiménez C., Sebastián E., Chillón M. C. (2013). MYD88 L265P is a Marker Highly Characteristic of, But Not Restricted To, Waldenström’s Macroglobulinemia. *Leukemia*.

[18] Poulain S., Roumier C., Decambron A. (2013). MYD88 L265P Mutation in Waldenstrom Macroglobulinemia. *Blood*.

